# Host immune responses in aged rhesus macaques against BBV152, an inactivated SARS-CoV-2 vaccine, and cross-neutralization with beta and delta variants

**DOI:** 10.3389/fimmu.2023.1161571

**Published:** 2023-04-28

**Authors:** Dilip R. Patil, Anita M. Shete, Pragya D. Yadav, Gajanan N. Sapkal, Gururaj R. Deshpande, Himanshu Kaushal, Sreelekshmy Mohandas, Siddharam Fulari, Rajlaxmi Jain, Ajay Kumar, Priya Abraham

**Affiliations:** Indian Council of Medical Research-National Institute of Virology (ICMR-NIV), Pune, India

**Keywords:** BBV152, aged rhesus macaques, immune responses, SARS-CoV-2, delta variant, beta variant

## Abstract

The magnitude and duration of immune response to COVID-19 vaccination in older adults are known to be adversely affected due to immunosenescence and inflammaging. The threat of emerging variants warrants studies on immune response in older adults to primary vaccination and booster doses so as to understand the effectiveness of vaccines in countering the threat of emerging variants. Non-human primates (NHPs) are ideal translational models, as the immunological responses in NHPs are similar to those in humans, so it enables us to understand host immune responses to the vaccine. We initially studied humoral immune responses in aged rhesus macaques employing a three-dose regimen of BBV152, an inactivated SARS-CoV-2 vaccine. Initially, the study investigated whether the third dose enhances the neutralizing antibody (Nab) titer against the homologous virus strain (B.1) and variants of concern (Beta and Delta variants) in aged rhesus macaques immunized with BBV152, adjuvanted with Algel/Algel-IMDG (imidazoquinoline). Later, we also attempted to understand cellular immunity in terms of lymphoproliferation against γ-inactivated SARS-CoV-2 B.1 and delta in naïve and vaccinated rhesus macaques after a year of the third dose. Following the three-dose regimen with 6 µg of BBV152 with Algel-IMDG, animals had increased Nab responses across all SARS-CoV-2 variants studied, which suggested the importance of booster dose for the enhanced immune response against SARS-CoV-2-circulating variants. The study also revealed the pronounced cellular immunity against B.1 and delta variants of SARS-CoV-2 in the aged rhesus macaques even after a year of vaccination.

## 1 Introduction

The COVID-19 pandemic has affected millions of people worldwide and posed a major challenge to the public health system with 754,367,807 confirmed cases including 6,825,461 deaths (Covid19.who.int). Global health experts and researchers have been working round the clock to develop a safe and effective COVID-19 vaccine. Within the course of the pandemic, many vaccine candidates have been developed and Pfizer/BioNTech, SII/Covishield, Janssen/Ad26.COV 2.S, Moderna/mRNA 1273, and Sinopharm COVID-19 vaccines received emergency user authorization in different countries (1). Although these vaccines have proved their potential related to safety and effectiveness in clinical trials, the question related to differences in immunity among young and old individuals remains unanswered. The study conducted in healthcare workers vaccinated with two doses of both Covishield and Covaxin vaccines indicated that the anti-spike antibody was significantly lower in age group >60 years with comorbidities or history of hypertension as compared with age group ≤60 years (2). Similarly, a study on anti-spike IgG antibody kinetics after two doses of the Pfizer–BioNTech vaccine revealed that there was a consistent decreasing trend in IgG positivity with increasing age (3). Poor immune response in older adults, due to immunosenescence and inflammaging, waning immunity with the time and threat of emerging variants, warrants studies on immune response to booster doses especially in the older adult population (4, 5). Representation of older adults in vaccine clinical trials is limited, and very few studies have been conducted to study the durability and magnitude of immune response in the older adult population.

Non-human primates (NHPs) are ideal translational models, as the immunological responses in NHPs are similar to humans, so it enables us to understand host immune responses to the vaccine. According to a recent report, a booster dose of the mRNA-1273 COVID-19 vaccine given to rhesus macaques indicated significantly increased levels of neutralizing antibodies (Nab) against all known SARS-CoV-2 variants of concern (VOCs) (6). Considering that the Nab responses gradually wane, we initially studied humoral immune responses in aged rhesus macaques employing a three-dose regimen of BBV152, an inactivated SARS-CoV-2 vaccine (7). Initially, the study investigated whether the third dose enhances the Nab titer against the homologous vaccine strain (B.1) and important VOCs (Beta and Delta) in aged rhesus macaques immunized with BBV152, adjuvanted with aluminum hydroxide (Algel 1)/aluminum hydroxide-imidazoquinoline class, a TLR7/8 agonist (Algel2). Later, we also attempted to understand cellular immunity in terms of lymphoproliferation against γ-inactivated SARS-CoV-2 B.1 and delta in naïve and vaccinated rhesus macaques after a year of the third dose.

## 2 Methodology

Eight female, aged >18 years, rhesus macaques at the animal house facility of the Indian Council of Medical Research-National Institute of Virology, Pune, were used for the study. Three groups (groups 1, 2, and 3) were formed, two animals in group 1 and three animals each in groups 2 and 3. The macaques were immunized by three consecutive intramuscular injections at 0, 14, and 62 days. Groups 1, 2, and 3 received SARS-CoV-2 vaccine 6 μg + Algel 1, 3 μg + Algel 2, and 6 μg + Algel 2, respectively. Blood samples were collected from the saphenous vein before immunization and 1 or 2 weeks after each immunization. The serum samples were separated and stored at -20˚C. The sera were tested using indigenous anti-SARS-CoV-2 monkey IgG ELISA, anti-SARS-CoV-2 S1 RBD IgG ELISA, anti-SARS-CoV-2 N protein IgG ELISA, and plaque reduction neutralization test (PRNT) to assess the immune response in rhesus macaques.

Anti-SARS-CoV-2 IgG and IgG subtyping ELISA was performed as described earlier (8). Briefly, plates were coated with 100 µl SARS-CoV-2 antigen (accession no. EPI_ISL_420546, SARS-CoV-2 B.1 variant isolated from a human throat swab sample in Vero-CCL-81 cells and γ-inactivated, 10 µg/ml) overnight at 4°C. Diluted rhesus macaque serum samples (100 µl, 1:100) were added and incubated at 37°C for 1 h, followed by the addition of goat anti-monkey IgG peroxidase-conjugated antibodies (1:15000) (Jackson ImmunoResearch, USA) for IgG ELISA and mouse anti-human IgG1, IgG2, IgG3, and IgG4 (1:400) (Invitrogen) for IgG subtyping ELISA and incubated at 37°C for 1 h. The plates were washed five times with a wash buffer (1× PBST) after each incubation step. Finally, the TMB substrate was added and the reaction was stopped using H_2_SO_4_ and absorbance was measured at 450 nm. The assay cutoff was OD > 0.2 and P/N > 1.5.

Additionally, anti-SARS-CoV-2 S1 RBD and N protein IgG ELISA was performed as described above with few modifications. Briefly, plates were coated with 50 µl of SARS-CoV-2 recombinant S1 RBD and nucleocapsid protein overnight at 4°C. Diluted rhesus macaque serum samples (50 µl, 1:50) were added and incubated at 37°C for 30 min, followed by the addition of goat anti-monkey IgG peroxidase-conjugated antibodies, and incubated at 37°C for 1 h. The plates were washed five times with a wash buffer (1× PBST) after each incubation step. Finally, the TMB substrate was added and incubated at room temperature in the dark for 10 min. The reaction was stopped using 100 µl stop solution (1N H_2_SO_4_), and absorbance was measured at 450 nm. The assay cutoff for both S1RBD and N protein ELISA was OD > 0.2 and P/N > 2.

The sera of BBV152 vaccinated rhesus macaques were subjected to the determination of 50% plaque reduction neutralization test with few modifications (PRNT50), as described earlier (9). Briefly, all the sera were heat inactivated and serially diluted (fourfold) starting at a dilution of 1:10. Furthermore, these samples were mixed with an equal amount of live SARS CoV-2 variants (B.1: EPI_ISL_420546, delta: EPI_ISL_2400521, beta: EPI_ISL_2036294) virus suspension containing 50–60 plaque-forming units (PFU) in 0.1 ml. After incubating the mixtures at 37°C for 1 h, each virus-diluted serum sample (0.1 ml) was inoculated onto a 24-well tissue culture plate containing a confluent monolayer of Vero CCL-81 cells. After incubating the plate at 37°C for 60 min, an overlay medium consisting of 2% carboxymethyl cellulose (CMC) with 2% fetal calf serum (FCS) in 2× MEM was added to the cell monolayer and the plate was further incubated at 37°C in 5% CO_2_ for 5 days. Plates were stained with 1% amido black for an hour. Antibody titers were determined as the highest serum dilution that resulted in >50 (PRNT_50_) reduction in the number of plaques.

To understand protective cellular immunity, both SARS-CoV-2-specific and generalized cellular immunity were evaluated. SARS-CoV-2-specific cellular immunity was investigated in terms of lymphoproliferation, as described previously (10). Briefly, peripheral blood mononuclear cells (PBMCs) were isolated from the blood samples of naïve (n = 5) and vaccinated (n = 5) rhesus monkeys by density gradient sedimentation. The vaccinated group included monkeys vaccinated with either 6 µg + Algel 1 or 6 µg + Algel 2. PBMCs at a density of 1 × 10^6^ cells/ml were cultured with γ-inactivated whole antigens of SARS-CoV-2 (B.1/D614G) (10 µg/ml), SARS-CoV-2 (B.1.617.2/Delta) (10 µg/ml), or phytohemagglutinin-M (PHA-M) (10 µg/ml) for 96 h in a humidified 37°C/5% CO_2_ incubator. At 80 h of incubation, 20 µl 5-bromo-2′-deoxyuridine (BrdU) labeling solution was added and samples were reincubated for another 16–18 h. Lymphoproliferation was evaluated using the ELISA method. The proliferation index (PI) was calculated as the ratio of optical density (OD) of stimulated and unstimulated cultures for each sample. The generalized cellular immunity was determined by examining the proportion of T cells, B cells, and NK cells in the peripheral blood of each rhesus monkey of the naïve (n = 5) and vaccinated (n = 5) study groups using flow cytometry. The blood samples were collected on day 464 post 3rd dose of vaccine. The sample preparation and analysis were done as previously described (10). Briefly, 0.1 ml of whole blood was surface-stained with appropriate fluorochrome-conjugated antibodies (BD Biosciences). Two sets of sample tubes were prepared, one for T cells (CD3-FITC, CD8-PE, CD4-APC) and another for B and NK cells (CD45-PerCP, CD3-FITC, CD20-PE, CD16+56). Following incubation of 30 min at 4°C, 2 ml of 1× RBC lysing buffer (BD Biosciences) was added and incubated at RT for 12 min. The sample tubes were centrifuged at 200×g for 5 min, and the supernatant was carefully discarded. The sample tubes were washed with 2 ml of washing solution, and the cell pellets were suspended in 500 μl wash buffer. Samples were acquired and analyzed on Attune NxT Acoustic flow cytometry (Thermo Scientific) using Attune NxT software on at least 50,000 lymphocyte events. The lymphocytes were gated based on forward and side scatter. CD3+ T cells were gated on the lymphocyte population to determine the percentage of CD4+ and CD8+ T cells. Similarly, CD45+ cells were gated on lymphocytes, and percentages of CD20+ and NK cells were determined.

The data were analyzed using GraphPad Prism 9.0 software (GraphPad Software, San Diego, CA). For the cellular immunity study, analysis was done between the groups by the non-parametric Mann–Whitney test. The statistical tests were two-tailed, and *P* < 0.05 was considered significant.

## 3 Results

**Figure 1:**
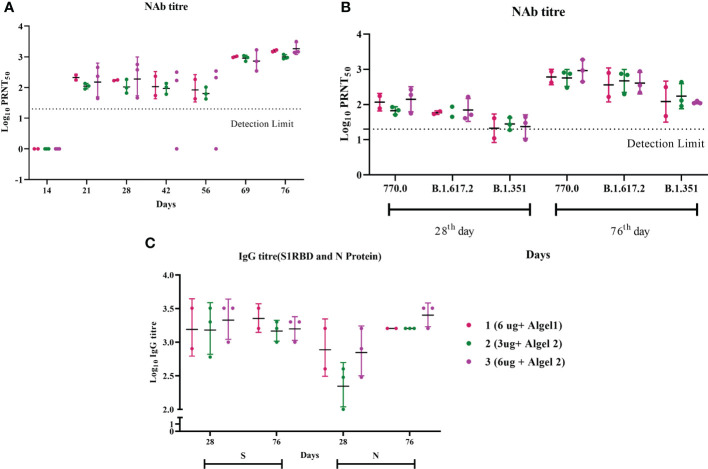
Antibody responses in vaccinated aged rhesus macaques. **(A)** Neutralizing antibody responses against the SARS-CoV-2 B.1 variant on different time points. **(B)** Neutralizing antibody responses against SARS-CoV-2 variants, B.1, delta, and beta on days 28 and 76. **(C)** S1-RBD and N protein IgG antibody response during the immunization with group 1, 2, and 3 vaccines.

During our earlier study involving young adult macaques, we followed a two-dose vaccination regimen on days 0 and 14, which elicited a very good IgG and neutralizing antibody response (8). However, when we tested the neutralizing antibody response 2 weeks after the second dose (day 28) in aged animals, it was comparatively less in all the groups (**Figure 1A**). The third dose was given on day 62, following which all animals showed a robust neutralizing antibody response. A clear boost in the GMT of Nab against the homologous vaccine strain was observed on day 76 (2 weeks post third dose) in all the groups (**Figure 1A**). The GMT Nab titers after the second dose (day 28) were 117, 67, and 141 for groups 1, 2, and 3, respectively, which sharply increased to 605, 567, and 922 for groups 1, 2, and 3, respectively, on day 76 (**Figure 1B**). These findings align with data from the phase II trial indicating that the antibody titers in the 6 µg with Algel-2 group were higher than those in the 3 µg with Algel-2 group (11). S1RBD and N protein ELISA also indicated anti-SARS-CoV-2 IgG antibodies in group 3 (1:1,587 and 1:2,539, respectively) as compared with groups 1 (1:2,262 and 1:1,600) and 2 (1:1,473 and 1:1,600) (**Figure 1C**). Subtyping analysis of the serum samples collected on day 76 from macaques revealed the presence of the IgG1 antibody except one animal from group 3 that was positive for IgG2 and IgG3 antibodies (**Supplementary Table 1**). We studied the cross-neutralization potential of the sera (day 28 and day 76: post 2nd and third doses, respectively) from the vaccinated macaques against delta and beta variants. We observed that the Nab titers increased four- to ninefold on day 76 as compared with day 28 against delta, beta, and homologous strain B.1 (**Figure 1B**). The GMT of Nab against delta variant were 59, 63, and 70 on day 28 for groups 1, 2, and 3, respectively, which sharply increased to 361, 468, and 406 on day 76. Similarly, the GMT of Nab against beta variant were 21, 28, and 23 on day 28 for groups 1, 2, and 3, respectively, which reached 121, 173, and 114 on day 76. However, when compared with the titers against a homologous strain (B.1), significant reduction in the Nab titers was observed against delta and beta variants (B.1 > Delta > Beta) (**Figure 1B**). The GMT ratio of B.1 to delta was 1.67, 1.21, and 2.27 for groups 1, 2, and 3, respectively, on day 76. Similarly, the GMT ratio of B.1 to the beta variant was 5, 3.2, and 8 for groups 1, 2, and 3, respectively, on day 76. In spite of the reduction, the titers against the variants remained above 100 on day 76, except for two animals against the beta variant.

**Figure 2:**
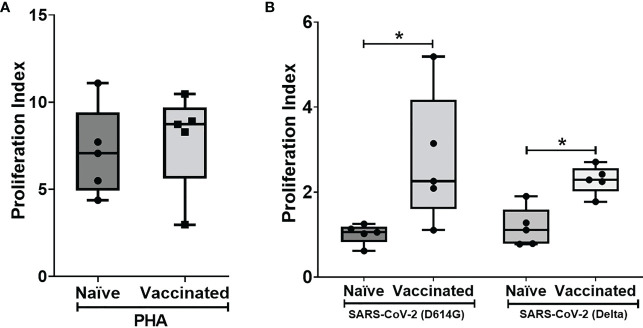
Lymphoproliferative responses to inactivated SARS-CoV-2 (B.1/D614G) and SARS-CoV-2 (B.1.617.2/delta): Peripheral blood lymphocytes from naïve (n = 5) and vaccinated (n = 5) monkey groups were cultured in the presence of **(A)** PHA and **(B)** inactivated SAS-CoV-2 (D614G/delta) for 96 h, and lymphoproliferation was measured by BrdU incorporation for the last 16–18 h using the Biotrak™ Cell Proliferation ELISA system. Data were analyzed between groups by the non-parametric Mann–Whitney test. The horizontal lines indicate the mean value. *P < 0.05 is considered statistically significant.

**Figure 3:**
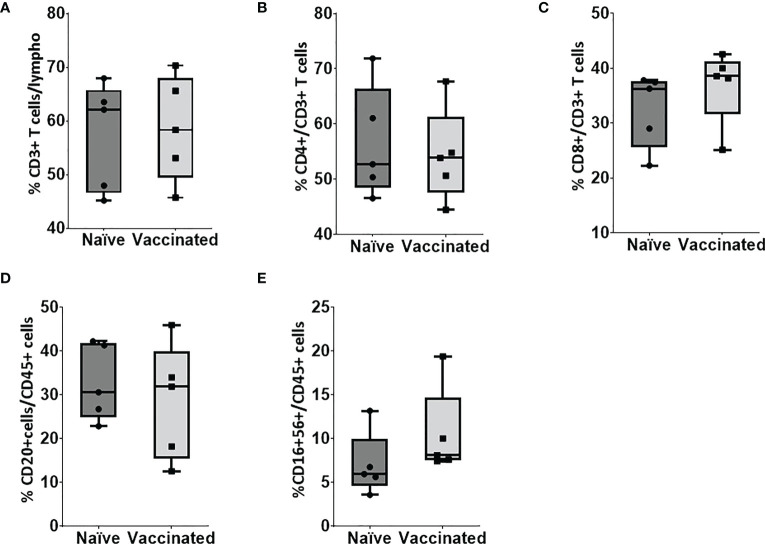
Flow cytometry analysis of lymphocyte subsets in peripheral blood. Blood samples of naïve (n = 5) and vaccinated monkey groups were surfaced stained with appropriate antibodies and analyzed using flow cytometry. Lymphocytes were gated based on forward and side scatter, and **(A)** CD3+ and CD45+ cells were gated on lymphocytes. **(B)** CD4+ and **(C)** CD8+ cells were gated on CD3+ cells whereas **(D)** B cells and **(E)** natural killer (NK) were gated on CD45+ cells. Horizontal lines indicate mean values.

*In vitro* cellular immune response was analyzed in the naïve and vaccinated rhesus monkeys in terms of lymphoproliferative response to inactivated SARS-CoV-2 (B.1/D614G) and SARS-CoV-2 (B.1. 617.2/delta), using PHA-M as a positive control. All cases showed a high proliferation index with PHA (naive PI mean ± SE, 7.14 ± 1.15; vaccinated PI, 7.87 ± 1.28) (**Figure 2A**). In response to SARS-CoV-2 (B.1/D614G), the group means of the vaccinated group (PI mean ± SE 2.76 ± 0.69, *P* < 0.0317) was found significantly high compared with the naïve group (PI mean ± SE, 1.01 ± 0.11) (**Figure 2B**). In response to SARS-CoV-2 (B.1.617.2/delta), the group means of the vaccinated group (PI mean ± SE 2.296 ± 0.15, *P* < 0.0159) were found significantly high compared with the naïve group (PI mean ± SE 1.17 ± 0.21) (**Figure 2B**). The assessment based on the surface markers of the gated lymphocytes from lysed whole blood showed no differences between the vaccinated and the naive group in terms of the percentage of cells expressing CD3, CD4, CD8, CD20, and CD16+56 (**Figure 3**).

## 4 Discussion

The neutralizing antibody titer is a well-established marker of protection in SARS-CoV-2 disease. A recent study in rhesus macaques revealed that passive transfer of a purified IgG antibody in rhesus macaques protected them against subsequent challenge with virulent virus. The Nab titers of 500 provided full protection whereas a titer of 50 was partially protective. The results indicated that even low levels of neutralizing antibody were partially protective (12). We observed a significant increase in neutralizing antibody titers post third dose. We could not conduct the challenge study with the variants in immunized macaques. However, recent studies with the three-dose inactivated vaccine regimen in macaques showed induction of a neutralizing antibody response (comparable with our study), which was cross protective *in vitro* and *in vivo* against the VOC (13, 14). These studies and our study indicate that the third dose of inactivated vaccine is important in boosting the antibody response so as to provide cross protection against the VOCs. In agreement with our findings, reduction in the titers against the delta and beta variants compared with the homologous strain has been observed in recently conducted studies on serum samples of vaccinees inoculated with mRNA as well as inactivated vaccines (3, 15). However, in spite of reduction in the titers, the neutralization potential against delta and beta variants has been established (15). Subtyping analysis of serum samples on day 76 revealed the predominance of the IgG1 antibody indicating a Th1-biased response. The finding is in agreement with a previous study showing Th1 skewed immune response against BBV152 in immunized animals (7).

The study revealed a significantly high proliferation of immune cells against both B.1/D614G and B.1.617.2/delta antigens in the vaccinated group compared with the unvaccinated naïve group. This indicated the presence of circulating SARS-CoV-2 B.1/delta-specific memory cells, responsible for the pronounced cellular responses in the vaccinated group, even after 1 year of vaccination. In addition, our data on cell phenotyping revealed no major changes in the generalized cellular immunity between the naïve and vaccinated groups, indicating that vaccines were well tolerated and no adverse effects or clinical complications were observed in the vaccinated group.

With unavailability of recently emerged isolates of VOCs XBB, BQ.1 CH 1.1, and XBB1.5, we could not study the cross protection potential of Covaxin (BBV152) against these variants. Our earlier studies have demonstrated the neutralization of Omicron variants with sera of Covaxin vaccinees (16–18). In addition to this, the hamsters immunized with Covaxin and further challenged with the Omicron variants, i.e., BA.1.1 and BA.2, demonstrated lesser virus shedding, low lung viral load, and less disease severity of lungs in the immunized groups compared with the non-immunized group (19). The data generated from these studies have proven the potential of Covaxin in cross protection against the Omicron variants. However, there is a need for future studies to assess the efficacy of Covaxin against these and emerging VOCs.

## 5 Conclusion

Here, we assessed host immune responses in aged rhesus macaques against a booster dose with BBV152, adjuvanted with Algel 1/Algel-2. Following the three-dose regimen with 6 µg of BBV152 with Algel-2, animals had increased Nab responses across all SARS-CoV-2 variants, which suggested the importance of a booster dose for the enhanced immune response against SARS-CoV-2 circulating variants. The study also revealed the pronounced cellular immunity against B.1 and delta variants of SARS-CoV-2 in the aged rhesus even after a year of vaccination.

## Data availability statement

The original contributions presented in the study are included in the article/**Supplementary Material**. Further inquiries can be directed to the corresponding author.

## Ethics statement

The animal study was reviewed and approved by Committee for Control and Supervision of Experiment on Animals, Ministry of Fisheries, Animal Husbandry and Dairying, Government of India.

## Author contributions

Plan and design of the study: PY, DP. Analysis of data, writing MS: PY, AMS, DP, HK, SM, GS, GD. Coordination for sample and data collection: SF, RJ. Laboratory investigation and report preparation: AMS, GD, SM, AK and HK. The authors PY, DP, and PA critically reviewed the manuscript. All the authors have reviewed and approved the final manuscript.
