# Supplementary material for: Host immune responses in aged rhesus macaques against BBV152, an inactivated SARS-CoV-2 vaccine, and cross-neutralization with beta and delta variants
Source: Front Immunol. 2023 Apr 28;14:1161571. doi: 10.3389/fimmu.2023.1161571 (PMC10175569; doi:10.3389/fimmu.2023.1161571)
Supplement: Supplementary file 1 [file Table_1.docx]

| **Groups** | **IgG** | | **IgG1** | | **IgG2** | | **IgG3** | | **IgG4** | |
| --- | --- | --- | --- | --- | --- | --- | --- | --- | --- | --- |
|  | **OD** | **P/N** | **OD** | **P/N** | **OD** | **P/N** | **OD** | **P/N** | **OD** | **P/N** |
| **Group 1**  **(6 ug+ Algel1)** | 1.241 | 15.264 | 0.445 | 3.97 | 0.075 | 0.37 | 0.237 | 0.61 | 0.086 | 0.44 |
|  | 0.613 | 9.245 | 0.302 | 2.57 | 0.327 | 1.47 | 0.265 | 0.96 | 0.187 | 0.59 |
| **Group 2**  **(3ug+ Algel 2)** | 0.923 | 14.000 | 0.526 | 3.70 | 0.616 | 0.86 | 0.117 | 0.25 | 0.445 | 1.37 |
|  | 0.829 | 13.003 | 0.216 | 2.57 | 0.075 | 0.50 | 0.136 | 0.95 | 0.128 | 1.64 |
|  | 0.744 | 10.635 | 0.242 | 1.50 | 0.460 | 0.89 | 0.223 | 0.43 | 0.321 | 1.07 |
| **Group 3**  **(6ug + Algel 2)** | 1.875 | 26.485 | 0.546 | 5.75 | 0.306 | 0.33 | 0.4 | 0.35 | 0.288 | 0.71 |
|  | 1.335 | 18.258 | 0.598 | 7.38 | 0.130 | 4.32 | 0.181 | 2.74 | 0.145 | 3.83 |
|  | 0.571 | 8.628 | 0.189 | 0.38 | 0.693 | 2.05 | 0.988 | 1.93 | 0.198 | 0.39 |

**Supplementary table 1:** The SARS-CoV-2 IgG antibody subtyping of the serum samples of the immunized macaques on day 76 (ie., two weeks post third dose).
